# Cardiomyocyte Proliferation and Maturation: Two Sides of the Same Coin for Heart Regeneration

**DOI:** 10.3389/fcell.2020.594226

**Published:** 2020-10-15

**Authors:** Ming-Tao Zhao, Shiqiao Ye, Juan Su, Vidu Garg

**Affiliations:** ^1^Center for Cardiovascular Research, The Abigail Wexner Research Institute, Nationwide Children’s Hospital, Columbus, OH, United States; ^2^The Heart Center, Nationwide Children’s Hospital, Columbus, OH, United States; ^3^Department of Pediatrics, The Ohio State University College of Medicine, Columbus, OH, United States; ^4^Department of Molecular Genetics, The Ohio State University, Columbus, OH, United States

**Keywords:** cardiomyocyte proliferation, cardiomyocyte maturation, cardiomyocyte hypertrophy, induced pluriopotent stem cells, cardiac stem cell therapy, cardiac regeneration

## Abstract

In the past few decades, cardiac regeneration has been the central target for restoring the injured heart. In mammals, cardiomyocytes are terminally differentiated and rarely divide during adulthood. Embryonic and fetal cardiomyocytes undergo robust proliferation to form mature heart chambers in order to accommodate the increased workload of a systemic circulation. In contrast, postnatal cardiomyocytes stop dividing and initiate hypertrophic growth by increasing the size of the cardiomyocyte when exposed to increased workload. Extracellular and intracellular signaling pathways control embryonic cardiomyocyte proliferation and postnatal cardiac hypertrophy. Harnessing these pathways could be the future focus for stimulating endogenous cardiac regeneration in response to various pathological stressors. Meanwhile, patient-specific cardiomyocytes derived from autologous induced pluripotent stem cells (iPSCs) could become the major exogenous sources for replenishing the damaged myocardium. Human iPSC-derived cardiomyocytes (iPSC-CMs) are relatively immature and have the potential to increase the population of cells that advance to physiological hypertrophy in the presence of extracellular stimuli. In this review, we discuss how cardiac proliferation and maturation are regulated during embryonic development and postnatal growth, and explore how patient iPSC-CMs could serve as the future seed cells for cardiac cell replacement therapy.

## Introduction

Heart failure has been a leading cause of morbidity and mortality in the world, affecting more than 40 million people. About 2% of adults will have heart failure and the percentage rises significantly for people over the age of 65 (Metra and Teerlink, 2017). The most common cause of heart failure is myocardial infarction (MI, also known as heart attack) which is frequently a consequence of coronary artery disease. During myocardial infarction, the blood flow decreases or stops to supply to a part of the heart, which leads to severe damage to cardiac muscle. Unlike skeletal muscle cells, adult cardiomyocytes are not capable of robust proliferation to regenerate the damaged myocardium. Instead, the damaged heart undergoes extensive remodeling to replenish the dead cardiomyocytes with fibrotic scars and pathological hypertrophy, which significantly impairs the normal cardiac function and eventually results in the development of chronic heart failure with either preserved ejection fraction (HFpEF) or reduced ejection fraction (HFrEF) (Swynghedauw, 1999; Borlaug and Paulus, 2011). Currently there is no effective curative regimen for heart failure, though the main treatment strategy focuses on improving the symptoms and preventing the progression of the disease. Regenerating the damaged cardiac muscle is the key point to restore the normal cardiac function in heart failure.

In the past few decades, numerous biomedical and bioengineering endeavors have been sought for heart regeneration (Laflamme and Murry, 2011). Therapeutic intervention strategies can be summarized into two categories: (1) stimulation of the proliferation of endogenous cardiomyocytes; and (2) transplantation of exogenous stem cells that can regenerate the damaged heart. Though amphibians and fish can efficiently regenerate the heart after injury (Poss et al., 2002), human adult heart has very limited ability for regeneration. The mammalian heart has transient regeneration potential shortly after birth (Porrello et al., 2011), and the turnover of human cardiomyocytes becomes less than 1% per year in adulthood (Bergmann et al., 2015). Therefore, the first regeneration strategy seems to be relatively elusive until major obstacles to resume the cell cycle entry of adult cardiomyocytes are overcome. In contrast, exogenous stem cell transplantation tends to be more promising with the recent discovery of human pluripotent stem cells (PSCs) including induced pluripotent stem cells (iPSCs) and embryonic stem cells (ESCs) (Thomson et al., 1998; Takahashi et al., 2007). While the existence of adult cardiac stem cells has been controversial (van Berlo et al., 2014; Chien et al., 2019), the beneficial effects of adult stem cell therapy could be attributed to an acute inflammatory-based wound-healing response that rejuvenates the infarcted area of the heart (Vagnozzi et al., 2020). Recent studies using human PSC-derived cardiomyocytes and cardiac patches have shown promising therapeutic treatment of myocardial infarction in large animal models, shedding light on the future cardiac regeneration therapies (Chong et al., 2014; Liu et al., 2018).

In this review article, we discuss the regulatory mechanisms that govern the proliferation of embryonic cardiomyocytes and the physiological maturation (hypertrophy) of postnatal cardiomyocytes. We explore the mechanistic insights of cardiomyocyte proliferation and maturation from the perspectives of cardiac differentiation of human PSCs. At the end, we envision the future use of human iPSC-derived cardiomyocytes for treating cardiovascular disease.

## Developmental Programs for Embryonic and Fetal Cardiomyocyte Proliferation

The human heart is the first functional organ to form during embryonic development. A fetal heartbeat can be detected as early as 6 weeks after gestation. Shortly after gastrulation and mesoderm commitment, early cardiac mesoderm which is marked by the transcription factor, MESP1, first appears at the posterior side of the embryo along with the primitive streak (Bondue et al., 2008). Cardiac mesoderm further migrates toward the anterior position of the embryo to form the reservoirs for the two cardiac progenitor cell populations: the first heart field (FHF) and the second heart field (SHF). The FHF is located at the cardiac crescent whereas the SHF is posterior to the crescent. The FHF progenitors generate the primitive heart tube and give rise to the left ventricle and parts of atria; the SHF progenitors migrate toward the primitive and looping heart tube and contribute to the right ventricle (RV), parts of the atria, outflow tract (OFT) which will form the aorta and pulmonary artery (Brade et al., 2013; Spater et al., 2014). The expression of the transcription factor, ISL1, distinguishes the FHF (ISL^–^) and SHF (ISL^+^) progenitors (Cai et al., 2003; Bu et al., 2009; Paige et al., 2015).

**FIGURE 1:**
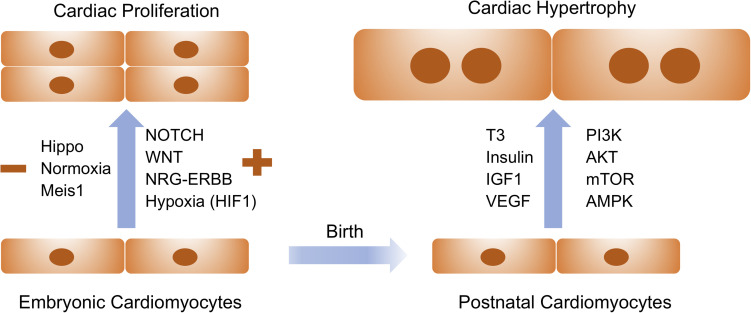
Extracellular signaling and intracellular pathways drive embryonic cardiac proliferation and postnatal cardiac hypertrophy. Signaling molecules listed on the left are positive and negative regulators for embryonic cardiac proliferation whereas those on the right are essential for physiological hypertrophy in postnatal cardiomyocytes. These pathways and molecules could be prospectively manipulated to stimulate cardiomyocyte proliferation and growth for therapeutic interventions of cardiovascular disease.

The *de novo* cardiomyocytes are derived either from the differentiation of early cardiac progenitors or from the proliferation of preexisting cardiomyocytes. Robust cardiomyocyte proliferation is necessary for proper formation of cardiac structures such as myocardial trabeculation and ventricular wall maturation. Multiple signaling pathways have been involved in the control of cardiomyocyte proliferation during this stage. The prominent pathways driving embryonic cardiac proliferation include NOTCH, Neuregulin (NRG), Hippo/Yap, IGF, and Wnt (Figure 1 and Table 1). The intensive crosstalk among these signaling pathways is embedded in the intercellular communication between the developing epicardium, myocardium and endocardium, and thus is highly coordinated to ensure the appropriate heart chamber growth in a spatiotemporal manner.

**TABLE 1:** Signaling pathways associated with cardiac proliferation and hypertrophic growth. Key references and species of these studies are listed.

Cardiac development	Signaling pathways	Species	Key references
Embryonic proliferation	NOTCH	Mouse	Grego-Bessa et al., 2007; Campa et al., 2008; Chen et al., 2013; Collesi et al., 2008; Luxan et al., 2013
Embryonic proliferation	NOTCH	Zebrafish	Zhao et al., 2014, 2019a
Embryonic proliferation	Hippo	Mouse	Heallen et al., 2011, 2013; Xin et al., 2011, 2013; von Gise et al., 2012
Embryonic proliferation iPSC-CM proliferation	WNT	Human/Mouse	Klaus et al., 2012; Buikema et al., 2013, 2020; Wang et al., 2016; Fan et al., 2018
Embryonic proliferation	Neuregulin-ERBB	Mouse	Gassmann et al., 1995; Lee et al., 1995; Meyer and Birchmeier, 1995; Erickson et al., 1997; Bersell et al., 2009; D’Uva et al., 2015
Embryonic proliferation	Neuregulin-ERBB	Zebrafish	Gemberling et al., 2015
Embryonic proliferation	Neuregulin-ERBB	Human	Polizzotti et al., 2015
Postnatal cell cycle exit	Cell cycle regulator	Mouse	Soonpaa et al., 1997; Engel et al., 2005; Pasumarthi et al., 2005; Sdek et al., 2011; Mahmoud et al., 2013; Nguyen et al., 2020
Postnatal cell cycle exit	Hypoxia	Mouse	Puente et al., 2014; Guimaraes-Camboa et al., 2015; Kimura et al., 2015; Tao et al., 2016; Nakada et al., 2017
Postnatal cell cycle exit	Hypoxia	Human	Ye et al., 2020
Physiological hypertrophy	Thyroid hormone	Mouse	Kinugawa et al., 2001; Belakavadi et al., 2010; Ferdous et al., 2020
Physiological hypertrophy	Insulin/IGF1	Mouse	Baker et al., 1993; Liu et al., 1993; Araki et al., 1994; Tamemoto et al., 1994; Reiss et al., 1996; Delaughter et al., 1999; Burks et al., 2000; Belke et al., 2002; McMullen et al., 2004; Kim et al., 2008

### NOTCH Signaling Pathway

In mammals, the NOTCH pathway includes 4 type I transmembrane receptors (NOTCH1-4) and 5 type I transmembrane ligands (JAG1, JAG2, DLL1, DLL3, and DLL4). The NOTCH receptors are heterodimeric latent transcriptional regulators and contain EGF-like repeats and Lin12/Notch (LNR) motif in the extracellular domain, and RBPJ associated molecule (RAM) motif, 7 ankyrin motifs, 2 nuclear localization signals, and a transactivation domain (TAD) in the intracellular domain. Upon ligand binding from the neighboring cell, NOTCH receptors are cleaved by the γ-secretase complex, resulting in the release of NOTCH intracellular domain (NICD). The NICD is then translocated to the nucleus, where it binds to RBPJ (CBF in humans) and MAML to form a trimeric complex that converts the RBPJ from a repressor to an activator and initiates the transcriptional activation of downstream target genes such as *Hey1* and *Hey2* (Siebel and Lendahl, 2017).

During embryonic heart chamber development, endocardial NOTCH signaling is essential for the trabeculation and compaction of ventricular myocardium (Grego-Bessa et al., 2007; MacGrogan et al., 2018). NICD/RBPJ activation stimulates BMP10 activity in the underlying myocardium which triggers the proliferation of cardiomyocytes through the inhibition of the cell cycle inhibitor p57 (Grego-Bessa et al., 2007). *Notch1* deletion in mouse embryos leads to reduced cardiomyocyte differentiation and impaired ventricular trabeculation, whereas exogenous *Bmp10* expression can rescue the phenotype in *Notch1* knockout (KO) embryos. In addition, endocardial deletion of the NOTCH1 negative regulator, FKBP12, leads to ventricular hypertrabeculation and non-compaction, in contrast to the hypotrabeculation in *Notch1* KO hearts (Chen et al., 2013). NOTCH signaling also controls the degradation of extracellular matrix (ECM) during the formation of endocardial projections through an antagonistic interaction with Neuregulin1 (NRG1) (Del Monte-Nieto et al., 2018).

Ectopic Notch activation promotes the cell cycle reentry and progression in isolated neonatal cardiomyocytes through transcriptional regulation of the cell cycle regulator cyclin D1 (Campa et al., 2008). In contrast, inhibition of Notch signaling blocks the proliferation of immature cardiomyocyte and induces apoptosis in mammals (Collesi et al., 2008). In zebrafish, Notch receptors are elevated in the endocardium and epicardium in response to ventricular amputation. Notch signaling is required for cardiomyocyte proliferation during zebrafish heart regeneration as suppression of Notch signaling impairs cardiac regeneration and induces scar formation at the amputation site (Zhao et al., 2014). Recent studies have demonstrated that endocardial Notch signaling stimulates cardiomyocyte proliferation in the regenerating zebrafish heart through upregulation of the Wnt signaling antagonists, Wif1 and Notum1b (Zhao et al., 2019a). In humans, mutations in NOTCH signaling components lead to a spectrum of congenital heart defects (CHDs). Pathogenic *NOTCH1* mutations are linked to severe forms of CHDs such as hypoplastic left heart syndrome (HLHS) and tetralogy of Fallot (Garg et al., 2005; McBride et al., 2008; Durbin et al., 2017; Page et al., 2019). Germline mutations in *MIB1*, a NOTCH pathway regulator, cause reduced Notch1 activity and lead to left ventricle non-compaction (LVNC) with abnormal ventricular myocardium development and trabecular maturation (Luxan et al., 2013).

### Hippo and Wnt Pathways

Hippo signaling is evolutionarily conserved in Drosophila and vertebrates for controlling tissue growth and organ size. The core components of Hippo pathway in mammals consist of a kinase cascade, MST1/2 (homology of Hippo in mammals) and LATS1/2, as well as the downstream effectors and transcriptional coactivators YAP and TAZ (Ma et al., 2019). The pathway activity modulates the dynamic shuttle of YAP/TAZ between the nucleus and cytoplasm. When the Hippo signaling is inactivated (OFF), the dephosphorylated YAP/TAZ are located in the nucleus and compete with VGLL4 for binding to TEADs. YAP/TAZ/TEAD complex then recruits other transcriptional factors to induce gene transcription. When the Hippo pathway is activated (ON), MST1/2 kinase phosphorylates LATS1/2 which further phosphorylates YAP/TAZ. The phosphorylation leads to the binding of YAP/TAZ to 14-3-3 and their cytoplasmic sequestration as well as degradation by poly-ubiquitination (Pan, 2010; Yu et al., 2015).

In mammals, Hippo pathway is an important regulator to restrain cardiomyocyte proliferation and control heart size (Wang et al., 2018). Genetic deletion of the Hippo pathway component *Salv* in cardiac lineage gives rise to enlarged heart at birth with increased proliferation of cardiomyocytes (Heallen et al., 2011). Similarly, embryonic knockout of the kinase *Mst1/2* or *Lats1/2* leads to embryonic lethality with massive cardiac overgrowth. Fetal deletion of the downstream effector *Yap1* results in lethal myocardial hypoplasia and reduced cardiomyocyte proliferation. In contrast, YAP1 activation stimulates cardiomyocyte proliferation both in culture and in the intact heart (von Gise et al., 2012). Nevertheless, cardiomyocyte size is normal in these Hippo knockout hearts, suggesting that Hippo signaling pathway suppresses cardiac proliferation but does not affect hypertrophic growth of cardiomyocytes (Heallen et al., 2020). In the postnatal heart, Hippo pathway activation prevents cardiac regeneration in response to injury as Hippo-deficient adult cardiomyocytes reenter the cell cycle and undergo cytokinesis in unstressed conditions (Heallen et al., 2013). In addition, suppression of Hippo pathway enhances adult cardiac regeneration and functional recovery after myocardial infarction and cardiac apex resection. For example, deletion of the Hippo pathway component *Salv* promotes a reparative genetic program with increased scar border vascularity, reduced fibrosis and enhanced ejection fraction after myocardial infarction. Cardiac specific deletion of the Hippo pathway effector *Yap* impairs neonatal heart regeneration whereas forced expression of *Yap* stimulates cardiac regeneration in adult heart (Xin et al., 2013). Gene therapy with short hairpin RNAs targeting *Salv* improves heart function when administered following myocardial infarction and ischemic heart failure (Leach et al., 2017), suggesting that Hippo pathway could serve as a promising therapeutic target for treating heart failure.

The regulatory roles of Hippo pathway in controlling cardiomyocyte proliferation and regeneration are executed through crosstalk with other signaling pathways (Wang et al., 2018). Active Wnt signaling is essential for silencing Hippo pathway. Cardiac-specific knockout of *Salv* in mice results in upregulation of Wnt target genes including *Sox2* and *Snai2* through the interaction between Yap and β-catenin (Heallen et al., 2011). When Hippo pathway is off, YAP enters the nucleus and forms a complex with TEA domain (TEAD) transcription factors and β-catenin/T-cell factor/lymphoid enhancer factor (Tcf/Lef), which binds to the promoters and regulates the transcription of genes associated with cardiac proliferation. In addition, the YAP/β-catenin complex also interacts with the insulin-like growth factor (IGF) signaling pathway. Elevated IGF signaling stimulates phosphoinositide-3-kinase (PI3K) which phosphorylates RACα serine/threonine-protein kinase (AKT). The phosphorylated AKT inhibits the phosphorylation of glycogen synthase kinase 3β (GSK3β) that is a member of the β-catenin destruction complex (Xin et al., 2011). Consequently, β-catenin is stabilized to drive the transcription of genes targeted by the YAP/β-catenin complex and promote cardiomyocyte proliferation and myocardial growth.

### Neuregulin-ERBB Signaling Pathway

Neuregulins (NRGs) are encoded by 6 individual genes *NRG1*, *NRG2*, *NRG3*, *NRG4*, *NRG5*, and *NRG6*, and are parts of the epidermal growth factor (EGF) family proteins (Mei and Nave, 2014). They can bind to the ligands of ERBB family of tyrosine kinase receptors (ERBB2, ERBB3, and ERBB4) through an extracellular EGF-like domain. The ERBB receptors consist of an extracellular ligand-binding domain, a transmembrane domain and a cytoplasmic tyrosine kinase domain. NRG1 binds to ERBB3 and ERBB4 (but not ERBB2) and causes the dimerization and activation of ERBB receptors, which subsequently phosphorylate their intracellular domains and provide docking sites for adaptor proteins such as Grb2/Shc for Erk activation and p85 for PI3K activation (Odiete et al., 2012).

NRG/ERBB signaling is essential for multiple aspects of early cardiac development. Disruption of *Nrg1*, *Erbb2*, or *Erbb4* leads to embryonic lethality by E10 *in utero* with lack of ventricular trabeculation and a mature ventricular wall (Gassmann et al., 1995; Lee et al., 1995; Meyer and Birchmeier, 1995). During embryonic heart development, *Nrg1* is expressed in the endocardium whereas *Erbb2* and *Erbb4* are expressed in the ventricular myocytes. *Erbb3* is expressed in the mesenchymal cells of the endocardial cushions that are essential for cardiac valve formation (Erickson et al., 1997). NRG1 promotes *in vitro* cell division of mononucleated ventricular cardiomyocytes through the receptor ERBB4 (Bersell et al., 2009). *In vivo* deletion of *Erbb4* suppresses cardiomyocyte proliferation whereas as overexpression of *Erbb4* enhances cardiac growth. Intriguingly, NRG1 injection in adult mice stimulates cardiomyocyte proliferation and promotes cardiac functional recovery after myocardial infarction. Recent studies reveal that NRG1 and its receptor ERBB2 can promote the regeneration of cardiac muscle cells in zebrafish, mice and infant heart tissue (Yutzey, 2015). NRG1 is induced in perivascular cells in response to adult heart injury in zebrafish. Myocardial overexpression of *Nrg1* enhances cardiomyocyte proliferation whereas *Erbb2* suppression disrupts cardiac regeneration in the injured heart. In unstressed zebrafish heart, NRG1 reactivation results in cardiomyocyte dedifferentiation, overt muscle hyperplasia and cardiomegaly (enlarged heart) through overgrowth of wall myocardium (Gemberling et al., 2015). In the mammalian heart, NRG1 co-receptor ERBB2 is essential for cardiomyocyte proliferation at both embryonic and neonatal stages. Reduced *Erbb2* expression causes diminished cardiac proliferation in the presence of NRG1. Constitutive overexpression of *Erbb2* results in an enlarged heart characterized by extensive cardiomyocyte hypertrophy, dedifferentiation and proliferation, whereas transient induction of ERBB2 promotes cardiomyocyte dedifferentiation and regeneration after myocardial infarction (D’Uva et al., 2015). Therefore, ERBB2 is required and sufficient to trigger postnatal cardiac proliferation and regeneration. Concomitantly, administration of recombinant NRG1 could improve myocardial function in response to injury and stimulate cardiomyocyte proliferation in cultured pediatric myocardium (<6 months old), suggesting a critical therapeutic window for regeneration therapy using NRG1 (Polizzotti et al., 2015).

## Regulation of Cardiomyocyte Maturation (Hypertrophy) After Birth

During mammalian embryonic and fetal development, heart growth is primarily driven by the cell division of cardiomyocytes. Shortly after birth, the increase of myocardial volume is transitioning to cardiac hypertrophy (an increase in the size instead of an increase in the number of cardiomyocytes) by which cardiomyocytes exit the cell cycle and become enlarged in both length and width to accommodate an increased cardiac workload (Nakamura and Sadoshima, 2018). In mice, cardiac regeneration capacity is retained 1 day after birth as the postnatal day 0 (P0) or P1 heart is capable of rejuvenating massive cardiac growth from the proliferation of preexisting cardiomyocytes in response to injury (Porrello et al., 2011). However, this regeneration ability diminishes by 7 days of age. In pigs, the neonatal heart is capable of regeneration after myocardial infarction in the first 2 days after birth, but the regeneration potential is lost soon after (Ye et al., 2018; Zhu et al., 2018). Interestingly, a thyroid hormone surge appears to activate the IGF/Akt pathway and initiate a transient but intense proliferative burst of binuclear cardiomyocytes (∼40% increase in cardiomyocyte number) in P15 mouse heart (Naqvi et al., 2014). Nevertheless, this phenomenon is contradicted by other studies where no evidence of an increase in cardiomyocyte number is observed in preadolescent mice (Alkass et al., 2015; Soonpaa et al., 2015).

The regulatory mechanisms governing the postnatal cardiac hypertrophy have been emerging in the past two decades. Extracellular signaling induced by growth hormones (thyroid hormone, IGF1 and VEGF) and mechanical stimuli (blood flow) are transduced to converge on a number of intracellular pathways which regulate the downstream gene expression, protein translation and metabolism that are essential for physiological hypertrophy in postnatal heart (Maillet et al., 2013). In the following section, we discuss the extracellular and intracellular signaling transduction mechanisms by which postnatal cardiomyocytes exit the cell cycle and transit to hypertrophic growth.

### Regulation of Cell Cycle Withdrawal in Postnatal Cardiomyocytes

Isotope tracing experiments show that the total number of cardiomyocytes is set within the first week after birth, despite of two waves of non-replicative DNA synthesis mainly contributing to cardiomyocyte multinucleation and nuclear polyploidization in the second and third postnatal weeks (Alkass et al., 2015). In humans, the turnover of cardiomyocytes is highest in early childhood but gradually decreases to less than 1% per year in adulthood (Bergmann et al., 2015). The mechanisms underlying the cell cycle arrest in postnatal cardiomyocytes have been of particularly interest to seek for potential therapeutic interventions for reactivation of cardiac proliferation.

Cell cycle regulators such as cyclins and cyclin-dependent kinases (CDKs) are playing important roles in the arrest of dividing cardiomyocytes (Ahuja et al., 2007). There is a critical cell cycle checkpoint called restriction (R) point in late G1 phase when cells must decide whether to advance through M phase or exit into the quiescent G_0_ stage. To advance cell cycle progression, cyclin D (cyclin D1, D2, or D3) must be accumulated and translocated to the nucleus, where it forms a complex with CDK4/6. The cyclin D-CDK4/6 complex then phosphorylates the Rb family (Rb, p107, and p130), leading to the release of E2F transcription factors which promote the synthesis of cyclin E (predominately expressed in the G1-S transition stage). Cyclin E interacts with CDK2 to form a cyclin E/CDK2 complex which drives additional Rb phosphorylation to push the cell cycle toward S phase and onward. E2F transcription factors are also essential for transcriptional regulation of genes that are involved in the exit to quiescent G_0_ phase.

The cell cycle positive regulators such as cyclin D1, CDK4/6, Rb, and E2F transcription factors are highly expressed in fetal cardiomyocytes but are significantly downregulated in neonatal and adult cardiomyocytes, coincident with the cell cycle arrest in postnatal cardiomyocytes (Kang et al., 1997; Flink et al., 1998). Constitutive overexpression of cell cycle regulators promotes DNA synthesis and increases multinucleation in cardiomyocytes, but completion of cytokinesis to generate new cardiomyocytes in adult hearts is relatively difficult to achieve. Transient overexpression of cyclin D1 or cyclin D2 stimulates DNA synthesis in adult hearts and results in infarct regression in response to cardiac injury (Soonpaa et al., 1997; Pasumarthi et al., 2005). Cardiac-specific deletion of p38 MAP kinase triggers the mitosis of neonatal cardiomyocytes whereas activation of p38 blocks fetal cardiomyocyte proliferation (Engel et al., 2005). Double knockouts of *Rb* and *p130* in the mouse heart result in the proliferation of adult cardiomyocytes by blocking their interactions with the heterochromatin protein 1 (HP1) (Sdek et al., 2011). A homeodomain transcription factor, Meis1, is recently identified as a critical regulator for cell cycle arrest in postnatal cardiomyocytes. Genetic deletion of *Meis1* extends the proliferation window of postnatal cardiomyocytes while stimulates the re-entry of mitosis in adult cardiomyocytes without detrimental influence in cardiac function. In contrast, overexpression of *Meis1* blocks neonatal cardiomyocyte proliferation and heart regeneration under stressed condition, possibly through the transcriptional activation of CDK inhibitors p15, p16, and p21 (Mahmoud et al., 2013). In addition, Meis1 interacts with its co-factor Hoxb13 to cooperatively regulate cardiomyocyte proliferation and maturation. Cardiomyocyte-specific deletion of *Hoxb13* defers the postnatal window of cardiac regeneration and reactivates cardiomyocyte cell cycle entry in adult heart, whereas double knockouts of *Meis1* and *Hoxb13* lead to widespread cardiomyocyte mitosis and improved left ventricle systolic function after myocardial infarction (Nguyen et al., 2020). Recently, the combinatorial expression of four cell cycle regulators *CDK1*, *CDK4, cyclin B1*, and *cyclin D1* was shown to be sufficient to induce cell division in post-mitotic cardiomyocytes across multiple species including mouse, rat and human (Mohamed et al., 2018). In acute or subacute myocardial infarction, 15–20% adult cardiomyocytes expressing these four factors can go through stable cytokinesis and improve cardiac function, implying a promising therapeutic application for cardiac regeneration.

In addition to the intrinsic control, cell cycle exit in cardiomyocytes is also tightly regulated by the extrinsic signals. Recent studies suggest that oxygen-rich environment is primarily responsible for the cell cycle arrest in postnatal cardiomyocytes (Puente et al., 2014). When newborns are exposed to normoxia (20–21% O_2_), reactive oxygen species (ROS), oxidative DNA damage and DNA damage response (DDR) markers significant increase in the heart. Intriguingly, reversal of normoxemia to hypoxemia conditions can extend the cardiac regenerative window and delay cell cycle withdrawal in neonatal cardiomyocytes. Genetic lineage tracing has identified rare hypoxic proliferative cardiomyocytes with smaller size, mononucleation and low oxidative DNA damage in neonatal hearts (Kimura et al., 2015). HIF1α is essential for the proliferation of hypoxic fetal cardiomyocytes during mid-gestation as loss-of-function of Hif1α leads to activation of ATF4 and p53 which inhibit cardiomyocyte proliferation (Guimaraes-Camboa et al., 2015). A transcription factor named Pitx2 potentially interacts with the Hippo pathway effector YAP to suppress the ROS levels. Genetic deletion of *Pitx2* results in compromised regeneration capacity in response to apex resection in neonatal heart (Tao et al., 2016), suggesting a crosstalk interaction between Hippo and HIF1α pathways in the regulation of cardiomyocyte regeneration. Furthermore, mice exposed to hypoxemia conditions display the reactivation of cardiomyocyte mitosis in the heart through inhibition of oxidative metabolism, decreased ROS production and oxidative DNA damage. One-week exposure to hypoxemia could induce robust myocardial regeneration from preexisting cardiomyocytes with decreased myocardial fibrosis and improved systolic function in response to myocardial infarction (Nakada et al., 2017). Similar beneficial effects of hypoxia on cardiomyocyte cell cycle activation are also observed in human myocardial tissue and iPSC-derived cardiomyocytes (iPSC-CMs), highlighting that hypoxia could serve as a potential therapeutic path forward toward human myocardial regeneration (Sadek and Olson, 2020; Ye et al., 2020).

### Physiological Hypertrophy of Cardiomyocytes in Postnatal Heart

During postnatal cardiac hypertrophy, cardiomyocytes become multinucleated, expand their contractile apparatus such as sarcomeres, and increase excitation-contraction coupling efficiency in order to maintain heart function upon an increased workload (Maillet et al., 2013). Physiological hypertrophy results in increased contractile function without interstitial fibrosis or cell death, which is distinct from pathological hypertrophy that leads to cardiomyopathy and heart failure (Nakamura and Sadoshima, 2018). Physiological hypertrophy is initiated by a number of extracellular stimuli which are transduced through multiple intracellular signaling pathways in order to modulate gene expression, protein translation and metabolic remodeling. These physiological signaling pathways include growth hormone, VEGF, thyroid hormone, insulin and IGF1. Note that pathological hypertrophy is regulated by numerous different extracellular signaling pathways and readers can refer to excellent reviews elsewhere (van Berlo et al., 2013; Nakamura and Sadoshima, 2018).

The active form of thyroid hormone T3 (3,5,3′-triiodothyronine) is essential for postnatal cardiac hypertrophy in mammals. Circulating T3 concentration in the blood surges (2,000-fold increase) in the second week after birth. T3 binds to the thyroid hormone receptors TRα and TRβ which are nuclear receptors. T3 binding results in the translocation of TR receptors into the nuclei, where they serve as transcriptional activators for initiating gene transcription (Brent, 2012; Mullur et al., 2014). In rodents, TR-responsive genes include upregulation of α-myosin heavy chain α-MHC (adult muscle isoform, *MYH6*) and sarcoplasmic reticulum Ca^2+^ ATPase (SERCA), and downregulation of β-myosin heavy chain β-MHC (fetal muscle isoform, *MYH7*) and phospholamban (*PLN*) in postnatal cardiomyocytes (Kinugawa et al., 2001; Belakavadi et al., 2010). T3-dependent gene expression in heart involves the crosstalk with retinoic acid receptors and is coordinated with histone modifications and non-coding RNAs to facilitate the removal of corepressors and recruitment of RNA polymerase II and coactivators (Mullur et al., 2014; Forini et al., 2019). Recent investigations uncover the forkhead box protein O1 (FoxO1) controls the enzyme Dio2 which is responsible for intracellular thyroid hormone metabolism, suggesting that FoxO1-Dio2 axis regulates T3-induced hypertrophic growth of neonatal cardiomyocytes (Ferdous et al., 2020).

Insulin and IGF1 are essential factors for initiating the physiological hypertrophy of postnatal cardiomyocytes. Insulin binds to the insulin receptor (IR) and activates the recruitment and phosphorylation of adaptor proteins such as insulin receptor substrate 1 (IRS1) and IRS2. IGF1 is structurally similar to insulin and binds to IR and IGF1 receptor (IGF1R). IR and IGF1R are tyrosine kinase receptors that activate PI3K-AKT-mTOR signaling pathway through the docking protein IRS1/IRS2, and RAS-RAF-MEK-MAPK signaling axis through the adaptor protein growth factor receptor-bound protein 2 (GRB2), respectively (Saltiel and Kahn, 2001; Taniguchi et al., 2006). Mice null for *Irs1/2* or *Igf1* display general growth retardation, and disruption of *Igf1r* results in perinatal lethality and a more severe growth deficiency (45% normal size) (Baker et al., 1993; Liu et al., 1993; Araki et al., 1994; Tamemoto et al., 1994; Burks et al., 2000). Cardiac-specific deletion of insulin receptor leads to reduced cardiomyocyte and heart size by 20–30% and persistent expression of the fetal β-myosin heavy chain isoform in mice (Belke et al., 2002). Overexpression of *Igf1* in the heart promotes cardiomyocyte proliferation and cardiac hypertrophy with enlargement of individual cardiac myofibers (Reiss et al., 1996; Delaughter et al., 1999). However, long-term local *Igf1* overexpression results in pathological hypertrophy with decreased systolic performance and increased fibrosis. Overexpression of *Igf1r* in the heart stimulates hypertrophic growth with an increase in cardiomyocyte size and enhanced systolic function, but without evidence of pathological hypertrophy (McMullen et al., 2004). Mice with cardiac-specific double knockouts of *Igf1r* and *Ir* appear normal in heart development but show resistance to exercise-induced hypertrophy, implying the essential role of IGF1 signaling in myocardial hypertrophic growth (Kim et al., 2008).

During postnatal cardiac growth, the vascular capillary network in the heart is proportionally expanded to supply sufficient nutrients and oxygen. Increased capillary activity leads to massive release of nitric oxide (NO) which results in the degradation of regulator of G-protein signaling 4 (RGS4), thus activating G protein-coupled receptor (GPCR) mediated downstream signaling pathways such as PI3K and mTOR to promote cardiac hypertrophy (Jaba et al., 2013; Oka et al., 2014). Reciprocally, myocardial hypertrophy also induces angiogenesis in the myocardium through activation of the intracellular signaling HIF1α and transcription factor GATA4. GATA4 is abundantly expressed in cardiomyocytes during early embryonic development and regulates the expression of cardiac-specific genes such as structural and contractile genes essential for hypertrophic growth. Cardiac-specific deletion of *Gata4* causes defects in cardiac hypertrophic growth and reduced density of myocardial capillary (Oka et al., 2006; Heineke et al., 2007). In contrast, cardiomyocyte-specific overexpression of *Gata4* increases capillary density in the myocardium. Furthermore, HIF1α is induced in the cell that is not receiving enough oxygen (hypoxia), and HIF1α trans-activates VEGF expression to stimulate angiogenesis (Forsythe et al., 1996; Semenza, 2014). VEGF is a critical factor in maintaining the myocardial capillary density during physiological cardiac hypertrophy through regulating angiogenesis (the growth of blood vessels from preexisting vasculature). Inhibition of VEGF signaling leads to suppression of capillary density and angiogenesis, which eventually promotes the transition from physiological hypertrophy to pathological heart failure during pressure overload (Shiojima et al., 2005; Izumiya et al., 2006).

## Harness Human iPSC-Derived Cardiomyocytes for Heart Regeneration

In the past few decades, cardiac stem cell therapy has been spearheaded as a potential treatment for myocardial infarction and heart failure. As there are no existing endogenous cardiac stem cells which can produce large number of *de novo* cardiomyocytes, patient-derived iPSCs possess great promise in cardiac regeneration (Murry and MacLellan, 2020). Human iPSC-derived cardiomyocytes (iPSC-CMs) are autologous and can be augmented for bulk production *in vitro* and generate billions of beating myocytes for repairing the damaged heart. Though precision generation of subtype-specific cardiomyocytes have been successfully achieved (Zhao et al., 2020), a major hurdle for the clinical application is the relative immature state of iPSC-CMs. Numerous strategies including *in vivo* transplantation and engineered 3D heart tissue with electromechanical coupling have been applied to improve the structural and functional maturation of iPSC-CMs (Karbassi et al., 2020). These approaches are designed to stimulate the maturation of iPSC-CMs by mimicking embryonic cardiomyocyte proliferation and postnatal hypertrophic growth during normal heart development. Here, we dissect how these cardiomyocyte maturation methodologies reflect the two perspectives of cardiac development: proliferation and maturation.

**FIGURE 2:**
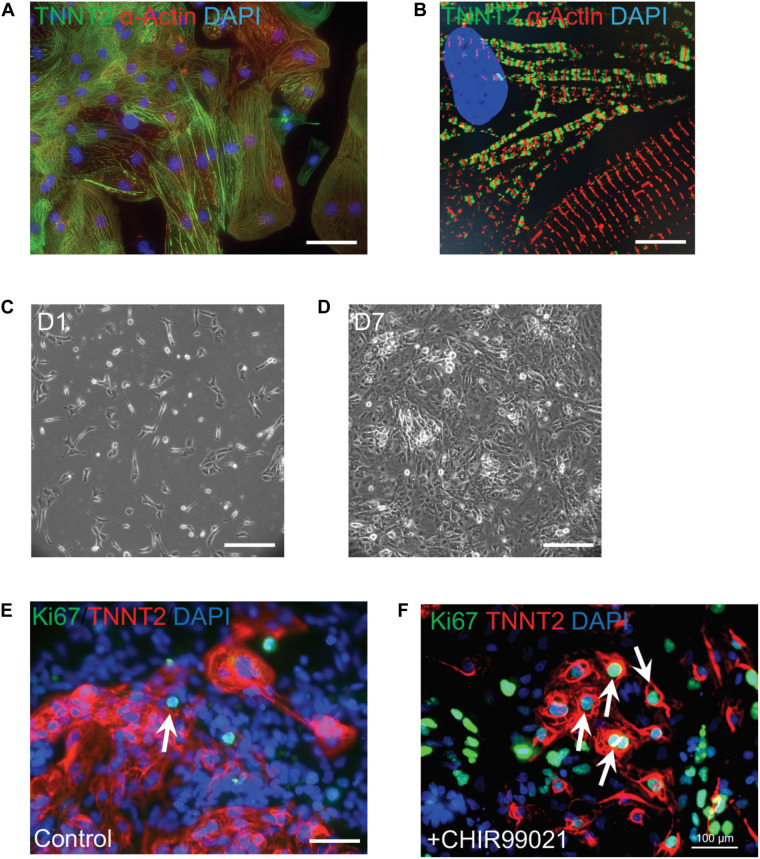
Robust proliferation of human iPSC-CMs is promoted by WNT signaling activation. **(A)** Typical morphology of human iPSC-CMs stained with antibodies against cardiac troponin T (TNNT2, green) and α-actinin (red). Nuclei were counterstained with DAPI (blue). **(B)** Zoom-in sarcomere structure of human iPSC-CMs with intercalated TNNT2 (green) and α-actinin (red). **(C,D)** Robust proliferation of human iPSC-CMs from Day 1 (D1,**C**) to D7 **(D)** in the presence of 2 μM of a WNT activator (CHIR99021). **(E,F)** Dramatic increase of dividing CMs is propelled by CHIR99021. Cells were stained with Ki67 (green) and TNNT2 (red). Nuclei were counterstained with DAPI (blue). Double positive cells (indicated by white arrows) are dividing cardiomyocytes. The percentage of Ki67^+^ TNNT2^+^ cells is increased in CHIR99021-treated iPSC-CMs **(F)** compared to the controls **(E)**. Scale bars: 10 μm **(B)**; 100 μm **(A,E,F)**; 200 μm **(C,D)**.

Current cardiac differentiation protocols mostly rely on manipulation of distinct signaling pathways to achieve robust cardiomyocyte generation (Protze et al., 2019; Zhao et al., 2020). In the early stage of directed differentiation, PSCs are induced to cardiac mesoderm and then cardiac progenitors by sequential modulation of WNT signaling pathway (Zhao et al., 2017). Cardiac progenitors are then directed to generate committed cardiomyocytes which resemble embryonic cardiomyocytes. There is a burst of cardiomyocyte proliferation to form ventricular chambers during embryonic heart development, which is modulated by the combinatorial interactions of signaling molecules such as NOTCH1, BMP10, NRG1, WNT, and VEGF (Foglia and Poss, 2016). An inhibitor CHIR99021 blocks GSK3-mediated degradation of β-catenin and leads to the nuclear translocation of β-catenin and activation of Wnt signaling target genes. Addition of CHIR99021 results in robust proliferation of mouse and human PSC-derived cardiomyocytes (Figure 2), consistent with the essential roles of the Wnt/β-catenin pathway for ventricular cardiomyocyte proliferation during embryonic development (Klaus et al., 2012; Buikema et al., 2013, 2020). In addition, Wnt signaling-induced cardiomyocyte proliferation is also observed in human neonatal cardiomyocytes and mouse adult cardiomyocytes (Wang et al., 2016; Fan et al., 2018). As NOTCH and Hippo pathways control cardiomyocyte proliferation through regulating Wnt/β-catenin pathway, manipulation of these pathway activities could boost the proliferation of early human iPSC-CMs to generate clinical-level magnitude for repairing the damaged myocardium.

*In vivo*, cardiomyocytes undergo structural and functional maturation accompanied with embryonic proliferation and postnatal hypertrophy. However, under current 2D differentiation protocols, human iPSC-CMs are relatively immature and resemble embryonic/fetal cardiomyocytes due to the absence of physical and environmental cues essential for the induction of physiological hypertrophy (Karbassi et al., 2020). Human iPSC-CMs are small (5–10 μm in diameter) and misaligned, compared to the rod-like shape (150 μm in length) and well-aligned adult cardiomyocytes (Snir et al., 2003; Yang et al., 2014). In addition, human iPSC-CMs generally do not have well-formed myofibrils and T-tubules, polyploidy, polarized intercalated discs or abundant mitochondria (Table 2). At the electrophysiological level, human iPSC-CMs beat spontaneously due to high levels of hyperpolarization-activated cyclic nucleotide-gate channel 4 (HCN4) present in the plasma membrane, whereas adult ventricular cardiomyocytes are usually quiescent unless receiving electric signaling from the depolarization of an adjacent cell. At the metabolic level, iPSC-CMs primarily rely on the energy release from glycolysis while in adult cardiomyocytes most (85%) of the energy supplies are from β-oxidation of fatty acids. At the gene expression level, human iPSC-CMs initially express the fetal isoform of myosin heavy chain (α-MHC, *MYH6*) and can gradually switch to the adult isoform (β-MHC, *MYH7*) dominant after long period of culture.

**TABLE 2:** Characteristic differences between human iPSC-CMs and adult cardiomyocytes, and multiple strategies to advance the maturation of iPSC-CMs.

Characteristics	Immature cardiomyocytes	Adult cardiomyocytes	Maturation strategies
Morphology	Circular, misaligned	Rod shape, well-aligned	Long-term culture, Substrate stiffness, 3D engineering, Cell patterning, Hormone treatment, Mechanical loading, Electrical stimulation, Metabolic intervention
Structure	Heterogeneous, flat	T-tubule, myofibrils	
DNA content	Diploid, mononucleated	Polyploid, multinucleated	
Gene expression	α-MHC	b-MHC	
Electrophysiology	Spontaneous beating	Quiescent	
Metabolic	Glucose	Fatty acid	
Cell cycle	Partially proliferative	Cell cycle arrest (G_0_)	

Despite the immaturity of iPSC-CMs, a number of approaches have been successfully used to advance iPSC-CMs toward a mature stage. Current state-of-the-art strategies include long-term culture, *in vivo* transplantation, 3D engineering heart tissue (EHT) coupled with electrical stimulation and mechanical stress, metabolic intervention and hormone treatment (Table 2). Of note, 3D EHT method appears to be most efficient as it can reconstruct the biophysical stimuli and intercellular crosstalk which are essential for the physiological hypertrophy of postnatal cardiomyocytes (Feric and Radisic, 2016; Scuderi and Butcher, 2017). Human cardiac tissues made from early-stage iPSC-CMs and supporting fibroblasts can advance to an adult myocardial-like state after a few weeks of 3D training under physical conditioning and electrical stimulation (Ronaldson-Bouchard et al., 2018). They show adult-like gene expression profiles, well-organized sarcomere structures, presence of transverse tubules, a positive force-frequency relationship and high-density mitochondria. Using a Biowire chip seeded with cell-hydrogel mixture, Zhao et al. (2019b) have constructed a platform which can generate atrial- and ventricular-specific cardiac tissue by combining the directed cell differentiation and electric field conditioning, which is very close to the simulation of distinct electric signaling in adult heart chambers.

## Conclusion and Perspectives

Extracellular signaling pathways governing the proliferation of embryonic and fetal mammalian cardiomyocytes have been discovered in the past few decades. Of these pathways, neuregulin and Hippo pathways have shown fascinating pre-clinical outcomes in stimulating endogenous cardiomyocyte proliferation in response to myocardial infarction. In addition, hypoxia-induced HIF1α pathway could be the next phase of therapeutic targets for myocardial regeneration, as HIF1α not only stimulates cell cycle reentry of postnatal cardiomyocyte but also promotes capillary angiogenesis to advance hypertrophic growth of cardiomyocytes. Concomitantly, human iPSC-CMs are promising exogenous sources for cardiac stem cell therapy due to the robust production and controlled progression toward adult-like phenotypes. Combined with CRISPR/Cas9-mediated genome editing (Doudna, 2020), patient iPSC-CMs are more defined and prone to genetic engineering, and exert advantages of beneficial clinical effects compared to adult stem cell therapy. Recent 3D heart printing and cardiac organoid technologies using iPSC-CMs have placed another hope for cardiac regeneration (Lee et al., 2019; Mills et al., 2019). Future thrives on innovative therapeutic strategies will be resorted to the knowledge that we are gleaning on the mechanistic control of cardiac differentiation, growth and regeneration.

## Author Contributions

M-TZ: conception and design, figure preparation, manuscript writing, and final approval of the manuscript. SY and JS: figure preparation and final approval of the manuscript. VG: manuscript writing and final approval of the manuscript. All authors contributed to the article and approved the submitted version.

## Conflict of Interest

The authors declare that the research was conducted in the absence of any commercial or financial relationships that could be construed as a potential conflict of interest.
